# Ultrasound-guided percutaneous tracheostomy: a risk-based protocol

**DOI:** 10.1186/s13089-024-00381-6

**Published:** 2024-06-03

**Authors:** Camilo Pérez, Daniel Ospina-Castañeda, Dormar Barrios-Martínez, Andrés Felipe Yepes

**Affiliations:** 1 Critical and Intensive Care Medicine Department, Hospital Universitario Fundación Santa Fe de Bogotá, Bogotá, Colombia; 2 Critical and Intensive Care Medicine Department, Hospital Universitario San Vicente Fundación, Medellín, Colombia; 3 School of Medicine and Health Sciences, Universidad del Rosario, Bogotá, Colombia; 4 Critical Ultrasound Group, Bogotá, Colombia

## Introduction

Tracheostomy is a common procedure in the intensive care unit (ICU) for patients on mechanical ventilation. It reduces the need for sedation, improves patient comfort, promotes early rehabilitation, and shortens the weaning process [1]. This procedure can be done using either the open or percutaneous approach [2]. In the past, open techniques were seen as the standard. However, these techniques tend to take longer and cost more without significantly reducing the risk of complications compared to percutaneous techniques. Open tracheostomies are now only used when a percutaneous technique is not possible due to anatomical difficulties of the neck, obesity, or neck length [3].

Percutaneous techniques can be guided by anatomical landmarks, bronchoscopy, or ultrasound [4]. Although there are various documented techniques, the most used currently is Ciaglia´s technique, which involves sequential dilation following the modified Seldinger technique until the stoma is created and the tracheostomy cannula is inserted using a guidewire [5]. The percutaneous approach has the advantage of being able to be performed at the bedside, by intensive care physicians instead of surgeons, and with less consumption of resources compared to open techniques [3].

**Fig. 1:**
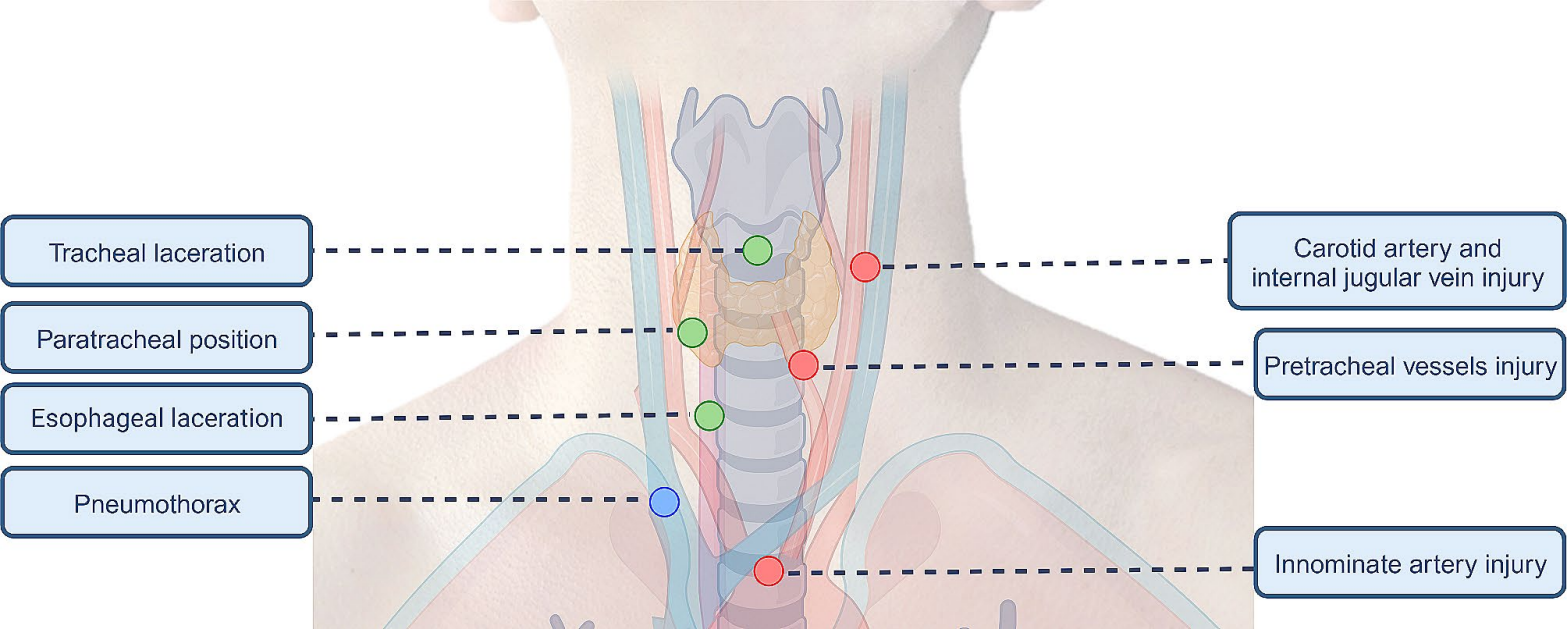
Anatomical relationships of complications associated with percutaneous dilatational tracheostomy. Establishing a safety margin for puncture and dilation is difficult based only on anatomical references and bronchoscopy due to the number of adjacent structures and their anatomical variations (Created with BioRender.com)

Although the complication rate after percutaneous tracheostomy is low, the complications that occur can be potentially lethal and include loss of airway, massive bleeding, pneumothorax, tracheal injuries, displacement, and occlusion of the cannula [6] (Fig. 1). The main factors associated with the development of complications are related to difficulties in identifying the anatomy of the airway. However, anatomical landmarks do not always predict the presence of anatomical variations, such as aberrant vascular trajectories, which may limit safety margins during puncture and dilation [7].

Using bronchoscopy or ultrasound guidance increases the likelihood of a successful puncture on the first attempt, simplifies the procedure, and reduces the risk of complications compared to guidance based on anatomical landmarks [7]. However, studies comparing the use of bronchoscopy and ultrasound have not been able to determine the superiority of one over the other for safety outcomes.

The ultrasound-guided technique offers advantages over bronchoscopy because it is more widely available in intensive care, requires fewer personnel to perform, and is considered equally safe. However, this technique has not yet been standardized and has a long learning curve, which may contribute to the variability observed in study results. This article proposes a risk mitigation protocol for performing ultrasound-guided percutaneous tracheostomy using the Ciaglia´s technique.

## Protocol

The protocol consists of 12 steps with three phases: planning, ultrasound-guided puncture, and screening. During the planning phase, the goal is to identify risk factors for complications and establish a safety margin for puncture and dilation. In the puncture phase, the aim is to minimize the risk of accidentally puncturing adjacent structures and ensure proper cannula placement. The screening phase is designed to promptly identify any complications that may arise after the procedure (Table 1).

**Table 1:** Ultrasound-guided percutaneous tracheostomy protocol

PHASE	STEP
Planning	**Step 1.** Identify innominate artery
	**Step 2.** Identify pretracheal structures
	**Step 3.** Identify lateral safety margins
	**Step 4.** Identify the cricoid cartilage
	**Step 5.** Measure the distance
	**Step 6.** Identify the airway axis
Puncture	**Step 7.** Position the endotracheal tube
	**Step 8.** Identify the position of the tube cuff
	**Step 9.** Real-time puncture
	**Step 10.** Identify the guidewire.
Screening	**Step 11.** Identify the position of the cannula
	**Step 12.** Rule out pneumothorax

**Fig. 2:**
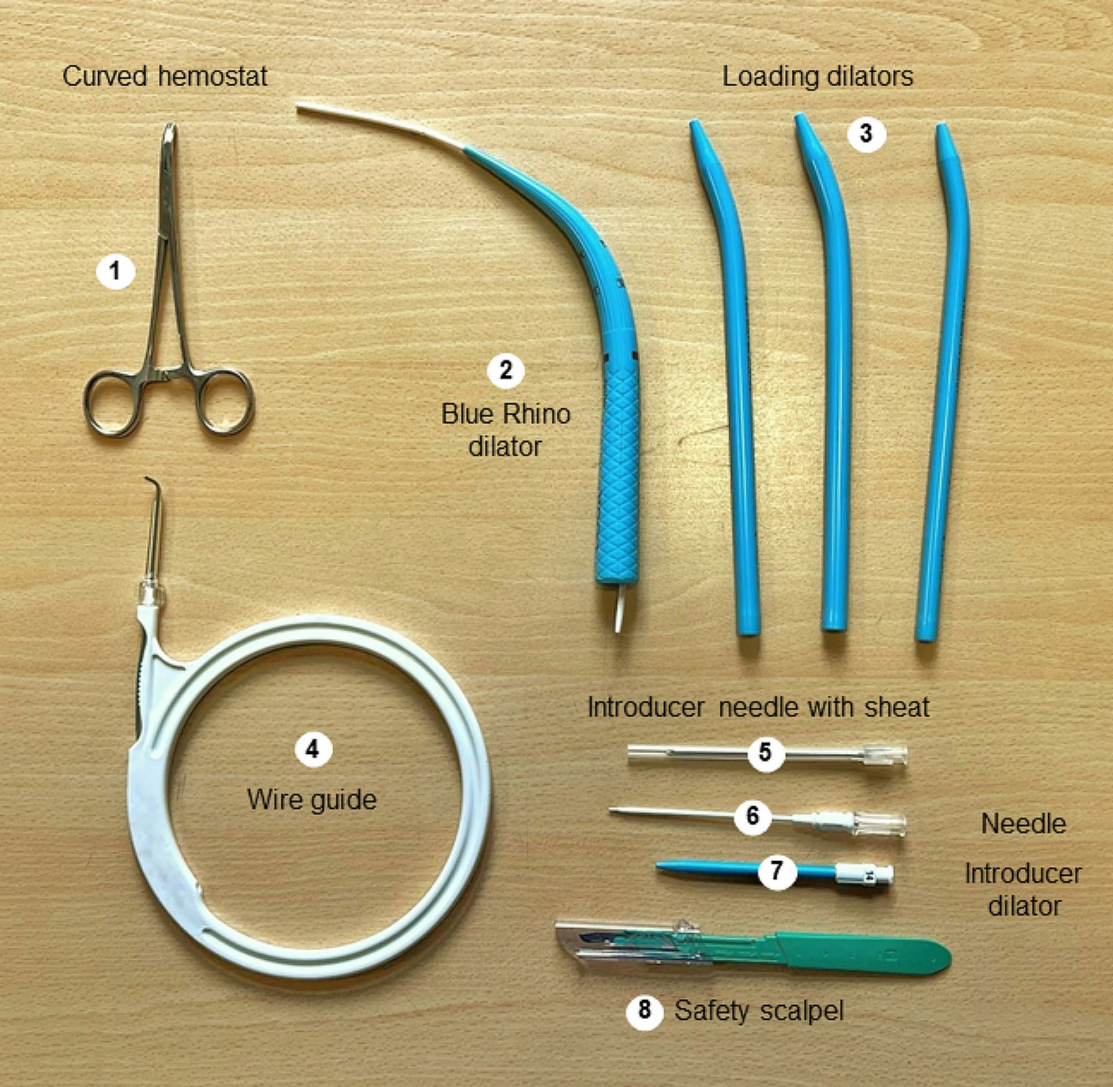
Tracheostomy kit

Prior to beginning the protocol, the team must ensure that there are no relative contraindications such as anatomical abnormalities, coagulopathy (platelets < 50,000 mm^3^, International normalized ratio (INR) > 1.5, activated partial thromboplastin time (aPTT) > 2 times control), intracranial hypertension, shock in the resuscitation phase, severe hypoxemia, and infections at the puncture site [1]. Once the team determines that the patient is eligible, they must explain the risks and obtain informed consent from the patient or their representative. A checklist should be prepared to ensure that necessary supplies are available, including sedation and paralysis medications, advanced airway equipment, and a tracheostomy tube of appropriate size. Additionally, the tracheostomy kit should be checked to include an introducer needle, metal guide, introducer dilator, guide catheter, and loading dilators (Fig. 2).

Ultrasound examination is performed with the patient lying on supine, the neck extended with a roll between the shoulders, and the head resting on a pillow. A linear and sector transducer is required, utilizing B-mode and Color Doppler.

### Step 1. Identify the course of the innominate artery

**Fig. 3:**
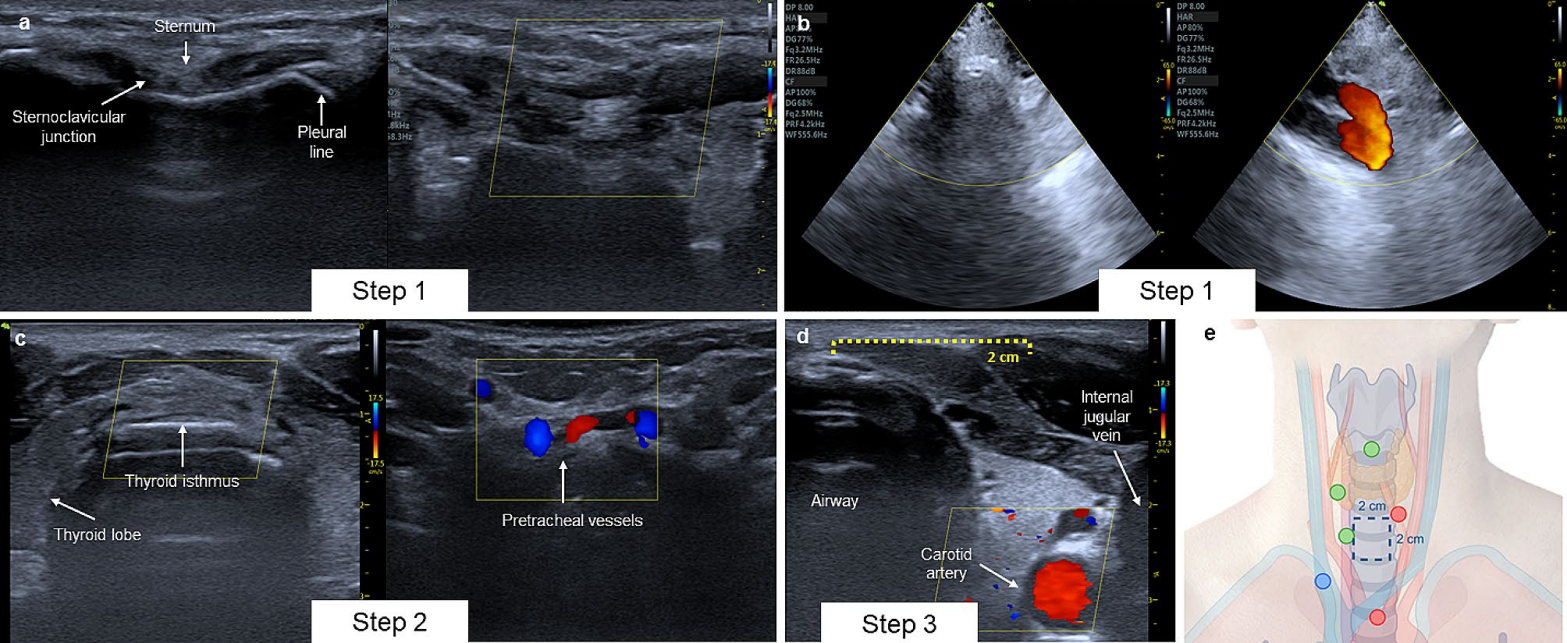
**A** Step 1: ultrasound exploration with linear transducer, absence of high-riding innominate artery. **B** Step 1 ultrasound exploration with sector transducer, innominate artery is identified with color Doppler. **C** Step 2: pretracheal structures identified with color Doppler along the transversal axis of the airway. **D** Step 3: Lateral margins are defined measuring the distance from the midpoint of the trachea to the lateral margin of the vessel. **E** The safety area is delimited (Created with BioRender.com)

Position the linear transducer in the sternal notch and identify the pleural line as a hyperechoic line between the acoustic shadow of the sternoclavicular junction. Position the color Doppler box in the center and scan caudally to cephalad until reaching the cricoid cartilage. The high course of the innominate artery may be present in up to 64% of healthy individuals [8]. By ultrasound, it is identified as a pulsatile structure that crosses anterior to the trachea above the sternal notch. It is also possible to identify it with the sector transducer by identifying the endothoracic bifurcation. If identified, the percutaneous procedure should be suspended and an open technique should be performed excluding the innominate artery (Fig. 3).

### Step 2. Identify pretracheal structures

Position the linear transducer on a short axis and scan from the cricoid cartilage to the sternal notch. Identify the tracheal rings by their inverted U-shaped morphology with a hyperechoic line in the posterior region that is accompanied by reverberation artifacts at the mucosa-air interface [9]. The thyroid lobes and isthmus are located lateral and anterior to the trachea, respectively. They are identified as isoechoic structures posterior to the sternohyoid muscle (Fig. 3) [10].

In 25% of patients, it is possible to identify pretracheal vascular structures that modify the safe puncture site [11]. To identify them, position the color Doppler box anterior to the trachea and identify vascular structures. It is recommended to apply gentle pressure to avoid the collapse of these structures and use pulsed Doppler to identify the phasic flow morphology of the veins and pulsatile flow of the arteries. Subsequently, it is recommended to measure the transverse diameter of the identified venous vessels [12]. Most of the pretracheal vascular structures identified are small-diameter veins that do not increase the risk of bleeding during the procedure. However, if arterial vessels or pretracheal veins with a diameter greater than 3.9 mm are identified, it is recommended to perform the open procedure due to the high risk of bleeding [13].

It is recommended to delimit in the short and longitudinal axis a safety area of 2 × 2 cm around the midline in the anterior tracheal region. If it is not possible to identify these limits, the open procedure should be considered (Fig. 3).

### Step 3. Identify lateral safety margins

In the percutaneous technique, there is a risk of performing an inadvertent paratracheal puncture, increasing the risk of injuring the common carotid artery and the jugular vein [14]. It has been described that in up to 15% of cases, the common carotid artery is located less than 10.5 mm away from the tracheal rings, which increases the risk of vascular injury during the procedure [15]. It is recommended to delimit a safety margin of at least 2 cm from the tracheal midline to the medial border of the great vessels. To do this, position the linear transducer in the short axis and explore with color Doppler from the cricoid cartilage to the sternal notch, measuring the safety area identified in step 2 (Fig. 3).

### Step 4. Identify the cricoid cartilage

Airway management complications are the second cause of death after performing a percutaneous tracheostomy. The most frequent are displacement of the tracheostomy tube, loss of the airway, and paratracheal position of the tube [16]. For this reason, it is recommended to have an advanced management plan for the emergent airway that includes surgical cricothyroidotomy in case of airway loss [17]. However, it has been shown that the ability to localize the cricothyroid membrane by trained personnel is limited [18].

Ultrasound can be used to identify the cricoid cartilage as an upper safety margin and as an anatomical reference to identify the cricothyroid membrane in case an emergency cricothyroidotomy is necessary. This approach has been shown to increase the probability of success on the first attempt compared to the technique under anatomical planes [19]. As part of the planning of the procedure, a short-axis high-frequency transducer must be explored until the cricoid cartilage is identified as a hypoechoic image with a horseshoe morphology and immediately above it the cricothyroid membrane, which is identified as a hyperechoic line with reverberation artifacts towards the air column [20]. Additionally, using ultrasound it is possible to measure the diameter of the subglottic airway, which has a strong correlation with the measurements taken by magnetic resonance imaging and can be useful in selecting the size of the tracheostomy tube [21]. To perform the measurement, position the linear transducer in the transverse axis at the level of the cricoid cartilage and measure the diameter of the air column as a line perpendicular to the midline of the airway.

**Fig. 4:**
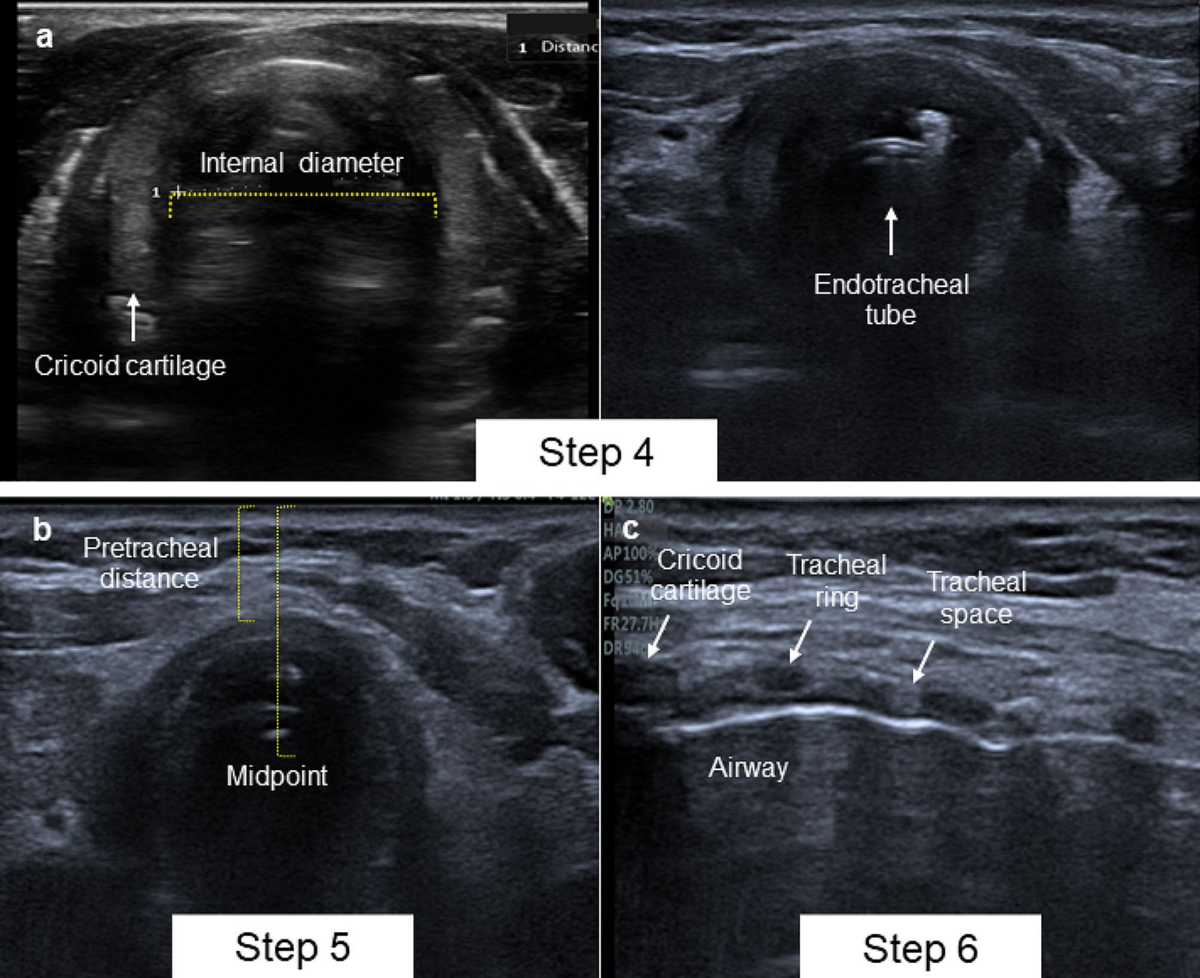
**A** Step 4: Cricoid cartilage and endotracheal tube visualization in transversal axis. **B** Step 5. Pretracheal and midpoint distance measurement. **C** Step 6 Airway longitudinal axis

### Step 5. Measure the pretracheal distance and the distance to the midpoint of the trachea

Puncture of the posterior tracheal wall increases the risk of tracheal perforation during percutaneous techniques [22]. Ultrasound can be used to measure the pretracheal distance and the distance to the tracheal midpoint to determine the margin for needle insertion. Additionally, measuring the pretracheal distance is helpful for selecting the cannula size in obese patients [23]. To measure, position the linear transducer in the transverse axis at the level of the cricoid cartilage, then descend to identify the tracheal rings and the safety area. Measure the pretracheal distance from the skin to the anterior portion of the tracheal ring, and the distance to the midpoint of the trachea from the skin to the midpoint of the air column.

### Step 6. Identify the longitudinal axis of the airway at the level of the cricoid

Ultrasound can be used to guide real-time puncture by identifying the anatomical structures of the airway, visualizing the endotracheal tube, and guiding the needle [24].

Initially, the longitudinal axis of the airway must be identified at the level of the cricoid. This can be done by locating the cricoid on the short axis using a linear transducer and making a 90° turn to identify the cricoid as a hypoechoic hump image. Tracheal rings can be identified as multiple rounded hypoechoic images that resemble a necklace of beads, accompanied by a posterior hyperechoic line resulting from reverberation between the mucosa-air surface [9].

### Step 7. Position the endotracheal tube

Loss of airway control can occur in the event of inadvertent puncture of the tube cuff, section of the endotracheal tube, and loss of the airway [25]. These complications can rapidly deteriorate patients with low functional reserve who are susceptible to loss of positive airway pressure and are at high risk of aspiration. Additionally, they can generate complications during dilation and increase the risk of tracheal lacerations [26]. For this reason, the position of the endotracheal tube must be guaranteed to reduce the risk of inadvertent puncture during the percutaneous technique [27].

**Fig. 5:**
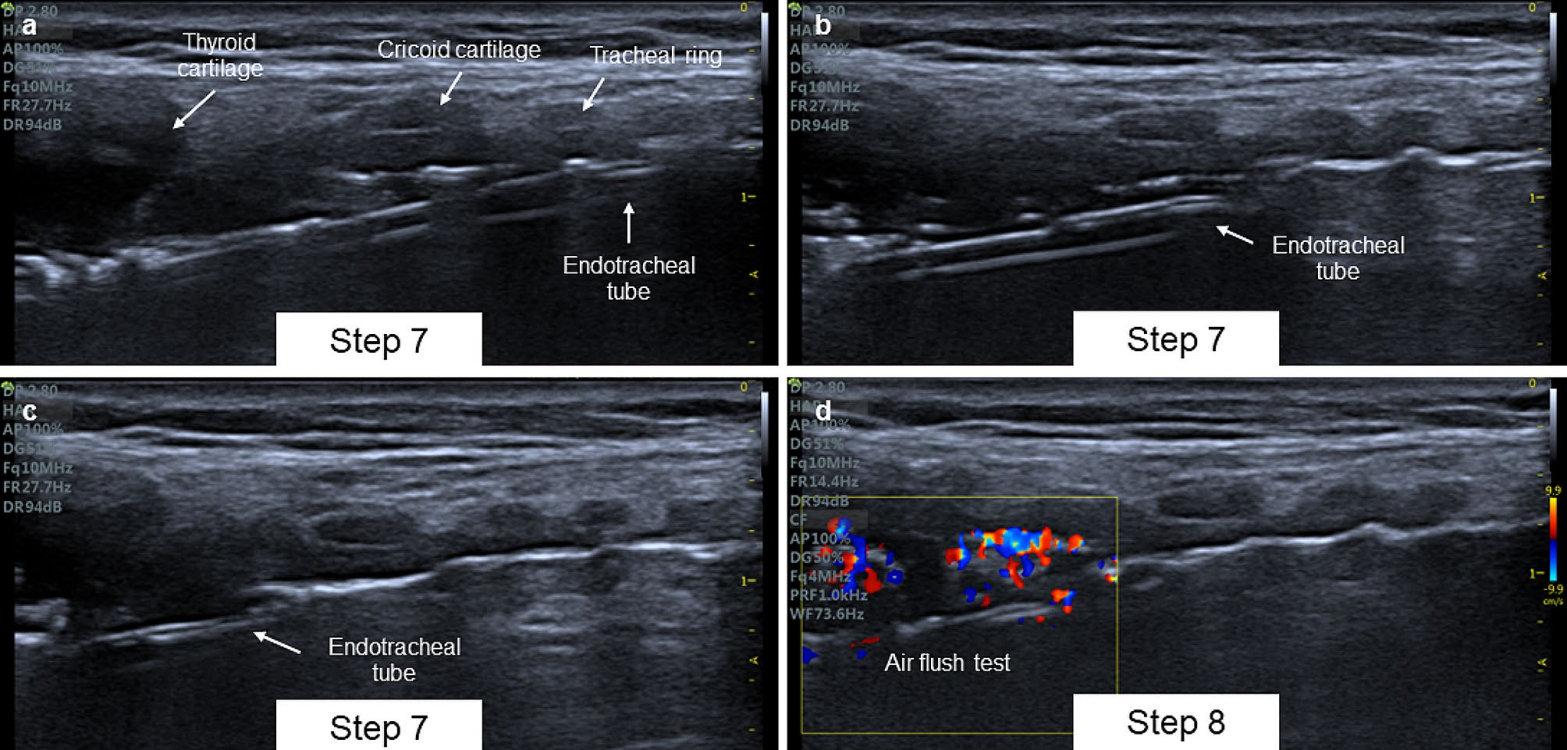
**A** Step 7: Endotracheal tube is visualized as two parallel hyperechoic line beyond the trachea. **B** Step 7: Real-time visualization of tube displacement. **C** Step 7: Distal end of the tube above cricoid cartilage. **D** Step 8: Air flush test

In the longitudinal axis of the airway, it is possible to identify the distal end of the endotracheal tube using ultrasound [28]. It is visualized through its lateral walls as two parallel hyperechoic lines posterior to the mucosa-air interface (Fig. 5). However, visualization may sometimes be difficult due to the air interface that separates the tracheal wall from the tube wall and prevents the transmission of the ultrasound wave. In these cases, it is recommended to ensure adequate cervical extension to move the distal end of the tube anteriorly and perform a tilting and rocking movement of the transducer to identify the end most proximal to the tracheal wall.

Once the distal end of the tube is located, it is recommended to remove it by viewing its position in real-time. The objective is to reposition the distal end of the tube until it is located between the thyroid cartilage and the cricoid cartilage to reduce the risk of puncture of the tube cuff.

### Step 8. Identify the position of the tube cuff

Tube cuff puncture can occur in up to 6.6% of cases with the percutaneous technique [29]. To reduce the risk of cuff puncture, its position superior to the cricoid must be guaranteed. Its identification is possible through the air flush test. To do this, place the color Doppler box at the distal end of the tube, deflate the cuff, and proceed to inflate it with 5 cc of air. With this maneuver, the flow should be visualized on color Doppler at the cuff location. If it is not visualized, the visualization of the distal end may be incorrect, so it is recommended not to continue removing the tube and repeat the steps described.

### Step 9. Real-time puncture

Using ultrasound to guide the puncture during percutaneous tracheostomy has been shown to increase the success rate on the first attempt and decrease midline deviation compared to the technique guided by anatomical landmarks [7]. As midline deviation increases, the force vectors exerted during dilation and insertion of the tracheostomy tube also increase, leading to a higher risk of complications such as kinking of the guidewire, difficulties during dilation, and tracheal lacerations and ruptures.

**Fig. 6:**
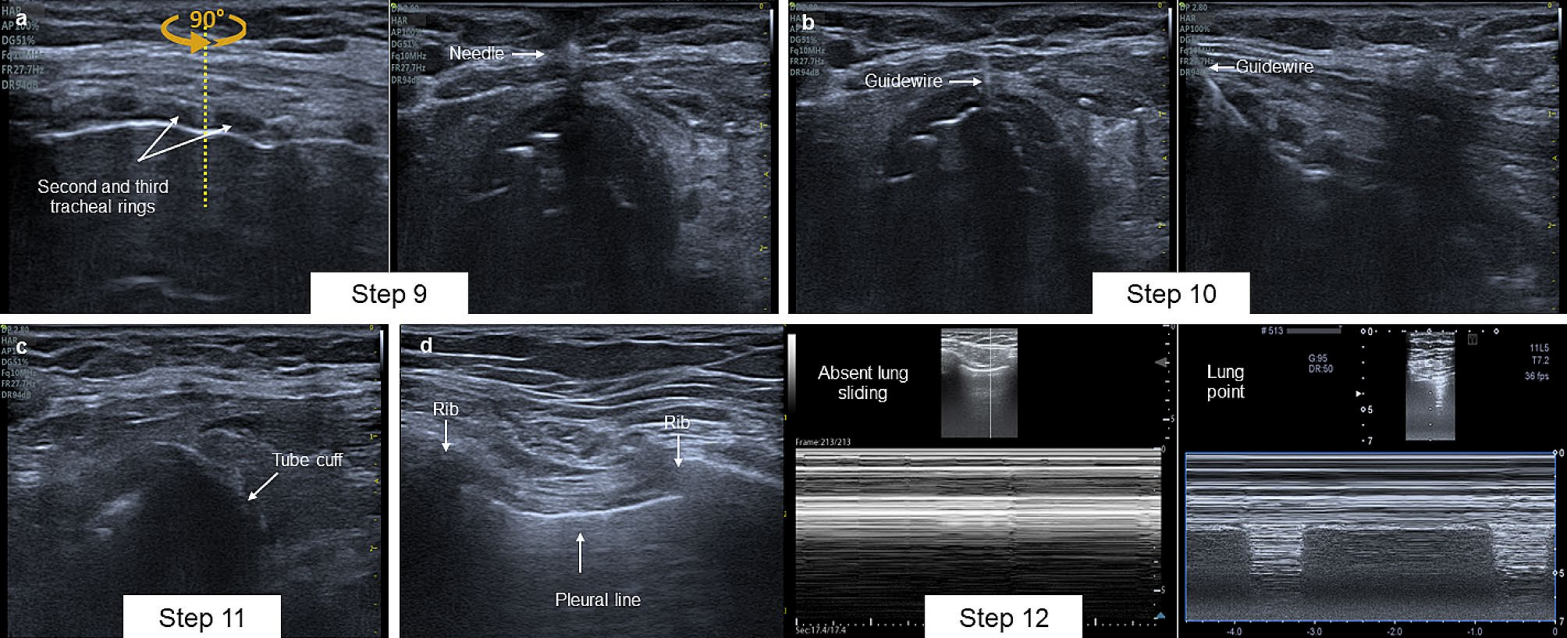
**A** Step 9: Transducer 90° rotation in longitudinal axis and real-time visualization of the needle. **B** Step 10: Guidewire path between second and third tracheal rings. **C** Step 11: Tube cuff. **D** Step 12: Assessment of lung sliding on B-mode and M-Mode, and lung point sign in M-Mode

Real-time puncture should be performed in the selected tracheal space based on defined safety margins. Initially, the tracheal space should be identified in the longitudinal axis and aligned with the midline of the transducer. Then, the transducer should be rotated 90° to obtain a transverse axis view. The puncture is performed by introducing the needle at 75% of the measured pretracheal distance. It is important to ensure that the acoustic shadow falls on the tracheal midline and then advance the needle until the measured distance to the tracheal midpoint is reached (Fig. 6).

### Step 10. Identify the path of the guidewire

Identifying the intratracheal path of the guidewire reduces the risk of pneumothorax, paratracheal dilations, a false route, or tracheal perforation [30]. After advancing the guidewire through the needle, it can be visualized as a hyperechoic line that crosses the pretracheal distance and the tracheal ring. To do this, position the linear transducer in the transverse axis over the puncture site and subsequently rotate 90° degrees until the entry site of the metal guide is identified in the longitudinal axis. In this way, it is possible to identify the intratracheal path of the guidewire and the entry site in the space between the two tracheal rings.

### Step 11. Identify the endotracheal position of the cannula

After sequential dilation, the tracheostomy cannula is introduced through the guidewire. Ultrasound can then be used to identify the endotracheal position of the cannula after inflating the cuff. With the linear transducer, identify the tracheal rings in the short axis. The cannula cuff is observed as a rounded hypoechoic image posterior to the tracheal rings. This allows for the identification of the cuff’s position and the exclusion of a paratracheal position for the tracheostomy cannula.

### Step 12. Rule out pneumothorax

Pneumothorax is a major complication after percutaneous tracheostomy [16]. The most common causes are injury to the posterior wall of the trachea, displacement of the cannula, inadvertent puncture, creation of a false route, paratracheal position of the cannula, and barotrauma induced by mechanical ventilation [31].

The final step of the protocol is to identify the presence of pneumothorax. The evaluation is performed with lung ultrasound using the linear transducer in B mode and M mode. It is recommended to explore both sides by scanning from the sternoclavicular junction to the mid-axillary line at the subcostal level. Lung sliding must be identified and, if absent, pneumothorax should be considered first. However, other causes of absent lung sliding include cannula obstruction, cannula malposition, and severe bronchospasm. For this reason, it is recommended that in the absence of lung sliding, the suspicion of pneumothorax be confirmed with the presence of the lung point sign or the absence of the lung pulse sign in M mode [32].

## Conclusions

The ultrasound-guided percutaneous tracheostomy technique can be standardized through a risk mitigation-based protocol. It is expected that the adoption of this protocol will reduce the incidence of vascular, mechanical, and airway management complications.

## Data Availability

Not applicable.

## Abbreviations

aPTT: activated partial thromboplastin time
ICU: intensive care unit
INR: International normalized ratio
